# The midline thalamic nucleus reuniens promotes compulsive-like grooming in rodents

**DOI:** 10.1038/s41398-025-03283-w

**Published:** 2025-02-25

**Authors:** Romeo Chen Wei Goh, Ming-Dao Mu, Wing-Ho Yung, Ya Ke

**Affiliations:** 1 School of Biomedical Sciences, Faculty of Medicine, The Chinese University of Hong Kong, Shatin, NT Hong Kong SAR, China; 2 Department of Neuroscience, City University of Hong Kong, Kowloon, Hong Kong SAR, China

**Keywords:** Neuroscience, Psychiatric disorders

## Abstract

Obsessive-compulsive disorder (OCD), a disabling and notoriously treatment-resistant neuropsychiatric disorder, affects 2–3% of the general population and is characterized by recurring, intrusive thoughts (obsessions) and repetitive, ritualistic behaviors (compulsions). Although long associated with dysfunction within the cortico-striato-thalamic-cortical circuits, the thalamic role in OCD pathogenesis remains highly understudied in the literature. Here, we identified a rat thalamic nucleus – the reuniens (NRe) – that mediates persistent, compulsive self-grooming behavior. Optogenetic activation of this nucleus triggers immediate, excessive grooming with strong irresistibility, increases anxiety, and induces negative affective valence. A thalamic-hypothalamic pathway linking NRe to the dorsal premammillary nucleus (PMd) was discovered to mediate excessive self-grooming behavior and render it a defensive coping response to stress, mirroring the compulsions faced by OCD patients. Given the close resemblance between this self-grooming behavior and the clinical manifestations of OCD, the results from this study highlight the role of NRe in mediating OCD-like compulsive behaviors. This can be attributed to NRe’s position at the nexus of an extensive frontal-striatal-thalamic network regulating cognition, emotion, and stress-related behaviors, suggesting NRe as a potential novel target for intervention.

## Introduction

Obsessive-compulsive disorder is one of the most prevalent neuropsychiatric disorders, functionally disabling hundreds of millions of individuals globally [1]. Characterized by persistent, intrusive thoughts or urges (*obsessions*) and repetitive, ritualistic behaviors (*compulsions*), OCD is often distressing and debilitating [2]. In addition to these hallmark features, previous literature has cognitive declines in response inhibition, executive planning, attentional set-shifting, and deficits in fear extinction [3–7]. Despite decades of research, the etiology and pathophysiology of OCD remain incompletely understood. Moreover, current treatment options show limited efficacy, with almost half of the patients resistant to first-line interventions [8].

Given the expansive growth in neuroimaging and optogenetic studies, current research extensively examines the cortico-striato-thalamic-cortical circuit’s role in the pathogenesis of OCD. The majority of research focus has been on the frontal cortices and striatum due to their crucial involvement in the generation of compulsive behaviors [9–11]. Although repetitive behavior is at the core of OCD, significant phenotypic heterogeneity exists among patients, with anxiety and distress being among the most common symptoms and triggers [12]. Thus, studies suggest that the neuroanatomical substrates of OCD might extend beyond cortico-striatal pathways, incorporating the roles of the thalamus, hippocampus, and amygdala [13]. Dysfunctions in five highly interactive neural circuits—the fronto-limbic, dorsal cognitive, ventral cognitive, ventral affective, and sensorimotor—are implicated in OCD symptomatology [12, 14]. Notably, the thalamus, once thought to primarily serve as a sensory relay to the cortices with occasional processing duties, is now recognized for its broader functions within these functionally segregated neural circuits. Indeed, neuroimaging studies have revealed structural alterations in the thalamus within the OCD population, highlighting a potential avenue for targeted treatment [15].

The thalamus comprises three groups of thalamic nuclei: the sensorimotor nuclei, the limbic nuclei, and a set of nuclei bridging these two domains, forming a sensorimotor-limbic continuum depending on their relative degree of involvement in sensorimotor or limbic functions [16]. The sensorimotor group contains principal nuclei that receive sensory or motor information via ascending pathways, which they transmit to cortical regions. Meanwhile, the limbic group—specifically the midline and intralaminar nuclei—plays a more significant role in affective and cognitive behaviors. In particular, a division of the ventral midline thalamus, including the reuniens (NRe), medial xiphoid, paraxiphoid, and rhomboid nucleus, has been extensively studied due to its strong reciprocal connections with the hippocampus and the medial prefrontal cortex (mPFC) [17]. The NRe is associated with diverse cognitive functions, especially those involving interactions with the hippocampus and mPFC, such as working memory, attentional processes, and behavioral flexibility [18–20]. Inactivation of the NRe produced spatial working memory deficits and severe behavioral perseveration, where rats persistently made incorrect choices during the correction phase of a task [18]. Moreover, the role of NRe in affective behaviors, such as fear, has been highlighted in recent literature. For instance, inactivation of the NRe disrupted the acquisition and expression of contextual fear memory and induced overgeneralized fear responses to novel environments [21–23]. The NRe is also an important hub for the consolidation and extinction of remote fear memories [24–26].

Additionally, aberrant activity in the NRe circuits altered the behavioral strategies adopted by rodents under stressful situations [27, 28]. Importantly, recent studies have started to identify key structural and functional similarities of the NRe in rodents and humans (often referred to as the midline thalamus), hence making careful extrapolation of data derived from animal models to humans justifiable [29–33]. Alterations in the function of the midline thalamus, including the NRe, and its connectivity with corticostriatal regions have also been implicated in the symptom severity of OCD in human patients [33–35]. In addition, the extensive reciprocal connections of the NRe with the prefrontal cortex and hippocampus, both of which are key nodes involved in the pathophysiology of OCD clinical symptoms [12–14], have further accentuated the currently overlooked potential of targeting the NRe as a novel avenue for targeted treatment [15]. Together, these findings have positioned the NRe, a component of the limbic thalamus, as a crucial node in the extensive cortico-hippocampal-thalamic network that regulates cognition, emotion, and stress-related behaviors [36].

In this study, we identified a previously uncharacterized role of the NRe in regulating persistent, repetitive self-grooming. Optogenetic activation of this thalamic nucleus elicited immediate and robust grooming behavior. Along with the persistence of this repetitive grooming, which can disrupt intake behaviors required for normal physiological functions, photoactivation of the NRe also induced place aversion and an anxiety-like phenotype. We further uncovered a thalamic-hypothalamic pathway that connects the NRe to the dorsal premammillary nucleus, which mediates such repetitive grooming behavior. Our findings thus necessitate a re-evaluation of the role of the ventral midline thalamus in the pathogenesis of OCD, an area that has not been extensively explored in current research.

## Materials and methods

### Animals

Adult male Sprague-Dawley rats weighing 260–320 g were used in this study. The animals, not strictly from the same litter, were bred and maintained by the Laboratory Animal Service Centre of the Chinese University of Hong Kong. The animals were grouply housed in ventilated standard cages and kept on a 12-h light/dark cycle. All experiments were performed during the light phase (09:00–19:00). The rats were handled in strict accordance with the university guidelines, with procedures approved by the Animal Experimentations and Ethics Committee to ensure animal welfare.

### Stereotaxic surgeries and optogenetic manipulation

Adeno-associated viruses (AAV), including AAV9-hSyn-eYFP (#50465), AAV9-hSyn-hChR2(H134R)-eYFP (#26973), and AAV9-hSyn-eNpHR3.0-eYFP (#26972) were purchased from Addgene (Watertown, USA). All viral titres were >5 × 10^12^ vg/mL. Rats were anesthetized with ketamine (75 mg/kg, i.p.) and xylazine (6 mg/kg, i.p.) and subsequently placed gently in a stereotaxic frame (Narashige, Tokyo). For intracerebroventricular (i.c.v.) infusion of adrenocorticotropic hormone (ACTH_1-24_, 4 µg), a stainless steel guide cannula was implanted bilaterally into the lateral ventricles (−0.72 mm A/P, ±1.70 mm M/L, −4.0 mm D/V, measured from bregma). For micro-injections, a Hamilton syringe (33-gauge) filled with AAV virus was lowered into NRe (−1.56 mm A/P, 0 M/L, −7.80 mm D/V, measured from bregma) following craniotomy. 0.2 µL of AAVs were infused at 10 nL/min, and the syringe was held in place for another 10 min to allow sufficient diffusion. The scalp incision was then sutured, and analgesics (buprenorphine, 0.05 mg/kg) were administered post-surgery to aid recovery. For optogenetic experiments, an optical fiber (200-µm core, NA = 0.37 m, Doric Lenses) was implanted 0.2 mm above the injection site. The fiber was then secured to the skull together with two stainless steel screws using dental cement. Rats were then allowed to recover for at least one week before behavioral testing. The location of virus expression and implants were confirmed postmortem. For ChR2 photostimulation, a 473 nm laser (10 ms, 25 Hz, Newdoon Technology) was delivered via an optic fiber cable (200-µm core, NA = 0.37, Doric Lenses). Laser power was measured to be 5 mW from the tip of the fiber. For eNpHR3.0 photoinhibition, a 589 nm laser (10 mW, Newdoon Technology) was continuously turned on throughout the session.

### Behavioral assays

For all behavioral tests, rats were habituated to the experimenter and testing apparatus for 30 min/day for at least three consecutive days before testing to reduce novelty-induced stress. The rats’ behavior in the test chamber (30 × 30 × 60 cm) was monitored and videotaped (Logitech, C922), and each test was repeated on different days to ensure reproducibility. For spontaneous grooming behavior, the rat was transferred from its home cage into the test chamber and its behavior was recorded for 10 min. For i.c.v. infusion of ACTH_1-24_ [37], ACTH was infused slowly via the cannula over a period of 60 sec, and the rat’s behavior was immediately recorded after the infusion for 10 min. For moisture-induced grooming [38], the rat was placed in an open circular pool (ϕ = 50 cm, height = 50 cm) filled with water at 25 °C. The water depth was set at 10 cm so the rat could stand freely with support. After 2 min, the rat was removed from the pool and placed immediately into the test chamber for another 10 min of videotaping. For body restraint-induced grooming, the rat was gently restrained in a black Plexiglas tube (ϕ = 5 cm, length = 25 cm) for 20 min and placed immediately into the test chamber for another 10 min of videotaping. For optogenetic experiments, the rats’ behavior was recorded following a 5-5-5 min photostimulation protocol, during which light pulses were delivered in the middle 5-min period. For the water-/food-restriction experiment, rats were placed on either a 24-h water or food deprivation, but not both, prior to testing. The videos were manually scored using Behavioral Observation Research Interactive Software (BORIS) [39].

### Definition of grooming behavior

Self-grooming behavior was recorded with digital video cameras (Logitech, C922) at 30 fps and manually scored using the BORIS software by two expert raters blind to the experimental conditions. The videos were replayed at 1/4 of the actual speed to aid accurate identification of the onset and offset of grooming behavior. The onset of grooming was defined as when the rat’s front paws reached its nose and started making elliptical paw strokes as defined in [9, 38, 40, 41]. The end of each grooming bout was defined as when the rat stopped grooming for at least 1 s or when the grooming behavior was interrupted by another behavior, such as rearing and locomoting. Parameters such as the number of grooming bouts, the duration of each bout, and the total duration spent grooming during the test period were evaluated. Social grooming was defined as when the rat demonstrated visible licking of the fur localized on the body trunk, shoulder region, or head of another conspecific, during which its forepaws were placed on the back or neck of the other rat, and its head showed bobbing movements indicative of licking motions [42].

### Real-time place preference/aversion assay

The rat was gently placed in a 50 × 120 cm rectangular test chamber, which was divided into two identical 50 × 60 cm compartments without additional contextual cues [43]. During the 15-min test session, one compartment was paired with an NRe-photostimulation while the other was without any photostimulation. The behavior of the rat was videotaped and subsequently analyzed by the ANY-Maze tracking software (Version 4.7, Stoelting CO) to obtain the percentages of time spent in each compartment. The behaviors of rats were also manually scored using BORIS to compute the latency at which rats switch from one compartment to another.

### Open field test

The rat was gently placed in the center of the open field (100 × 100 × 40 cm) and allowed for free exploration for 10 min. Next, the rat was subjected to optogenetic stimulation of NRe for 5 min. After the laser was switched off, the rat’s activity was recorded by a video camera for another 10 min. Parameters, including total freezing duration and time spent in the center and periphery zone, were analyzed by the ANY-Maze tracking software (Version 4.7, Stoelting CO).

### Elevated plus maze

The self-made elevated plus maze apparatus was made up of two open arms (50 × 10 cm) and two closed arms (50 × 10 × 40 cm) arranged in a “plus” shape and elevated 50 cm above the floor. The rat was gently placed at the junction of the open and closed arms while facing toward the opposite open arm. The rat was allowed 10 min of exploration, and its behavior was videotaped. Parameters, including the number of entries in the open/closed arms, total distance traveled and duration in the open/closed arms, and total freezing duration, were analyzed and quantified by the ANY-Maze tracking software (Version 4.7, Stoelting CO).

### Histology

Rats were anesthetized with a ketamine/xylazine cocktail and transcardially perfused with PBS, followed by 4% PFA in PBS. The brain was retrieved and incubated in 4% PFA at 4 °C overnight and for 48 h in a 30% sucrose solution. Fixed samples were sectioned into 30-μm coronal sections on a cryostat (ThermoFisher). For c-Fos staining, rats were sacrificed 60 min after behavioral testing. For immunostaining, free-floating brain sections were blocked in 5% normal goat serum in PBS with 0.3% Triton X-100 for 2 h and then incubated with anti-c-Fos primary antibody (1:1000; Cell Signaling Technology) at 4 °C overnight. After thorough rinsing in PBS, the sections were incubated with goat anti-rabbit IgG secondary antibody (1:1000, Invitrogen) in the blocking solution. Next, the sections were rinsed in PBS again and counterstained with DAPI before mounting. Microscopic images were acquired on a confocal laser scanning microscope (C1, Nikon).

### Statistical analysis

Statistical analysis was performed in GraphPad Prism 10. Data were presented as mean ± standard error of the mean (SEM) unless otherwise specified. Student’s t-test was used to compare two independent or paired groups. One-way ANOVA was used to compare multiple groups of samples, followed by the Tukey post hoc test for pairwise comparisons. Wilcoxon signed-rank test was used to compare non-parametric data. Methods used for each analysis are mentioned in the main text, as well as corresponding figure legends. The cutoff of significance was set at *P* = 0.05.

## Results

### Activation of thalamic NRe induces repetitive and excessive grooming behavior

**Fig. 1:**
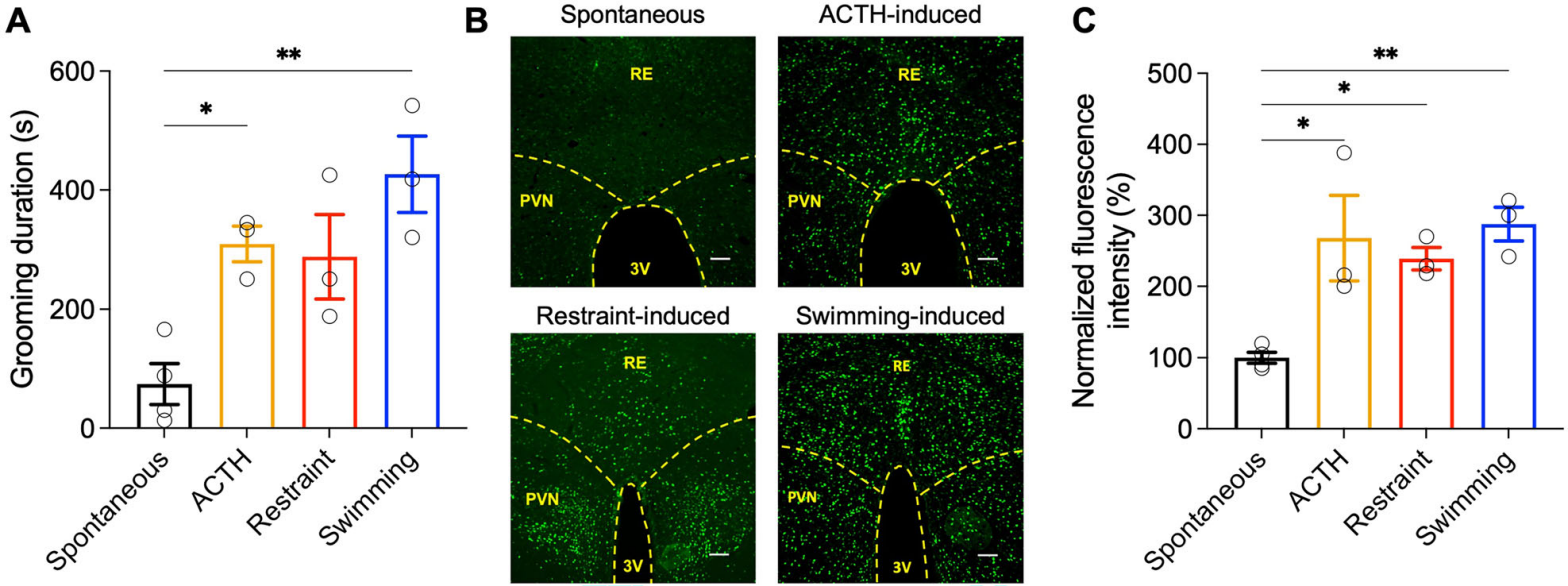
Activation of thalamic nucleus reuniens in different induced grooming behaviors in rats. **A** Rats subjected to ACTH i.c.v. infusion, body restraint, or fur-moistening through swimming (*n* = 3 in each group) demonstrated increased grooming compared with spontaneous grooming level (*n* = 4). One-way ANOVA: effect of treatment on grooming duration: F(3,9) = 9.282, *p* = 0.0041. Post-hoc Tukey HSD test; *p < 0.05, **p < 0.01, compared with spontaneous condition. **B** Representative images showing c-Fos^+^ neurons in the NRe following various grooming-inducing models. Scale bar, 100 µm. **C** c-Fos immunostaining revealed increased activation of NRe neurons after rats exhibited elevated grooming behavior induced by various stressors. One-way ANOVA: effect of treatment on c-Fos expression level: F(3,9) = 8.660, *p* = 0.0051. Post-hoc Tukey HSD test; *p < 0.05, **p < 0.01, compared with spontaneous condition. All data are presented as mean ± SEM.

To develop a model that emulates the repetitive behaviors seen in OCD patients, we subjected rats to a variety of stressors, including physiological, physical, and emotional, and examined their self-grooming behavior, a repetitive behavior commonly observed in rodents that serves as an adaptive response to stress [38]. The physiological stressor involved i.c.v. infusion of ACTH_1-24_, which is known to activate the hypothalamic-pituitary-adrenal axis [44, 45]. Physical stress was applied by wetting the rats’ fur through swimming, simulating a grooming trigger, and emotional stress was introduced by restraining the rats in a tube, a situation that induces anxiety [38, 41]. Upon exposure to either one of these stressors, an increase in grooming duration was observed in comparison to baseline, spontaneously occurring grooming (Fig. 1A). To illustrate, control, non-stressed animals displayed grooming for an average of 74.3 ± 34.5 s during a 10-min observation period. In contrast, ACTH-infused rats showed a grooming duration of 309.3 ± 29.9 s, restraint-stressed rats groomed for 287.7 ± 80.0 s, and fur-moistened rats for 426.7 ± 64.2 s (One-way ANOVA: effect of treatment on grooming duration: F(3,9) = 9.282, *p* = 0.0041). Corresponding to these behavioral changes, we detected robust c-Fos expression, an early indicator of neuronal activation, in the NRe. The swimming-induced model exhibited the most pronounced NRe activation, with a 288 ± 24% increase relative to spontaneous grooming, followed by ACTH-induced (268 ± 60%) and restraint-induced activation (242 ± 15%) (One-way ANOVA: effect of treatment on c-Fos expression level: F(3,9) = 8.660, *p* = 0.0051) (Fig. 1B).

**Fig. 2:**
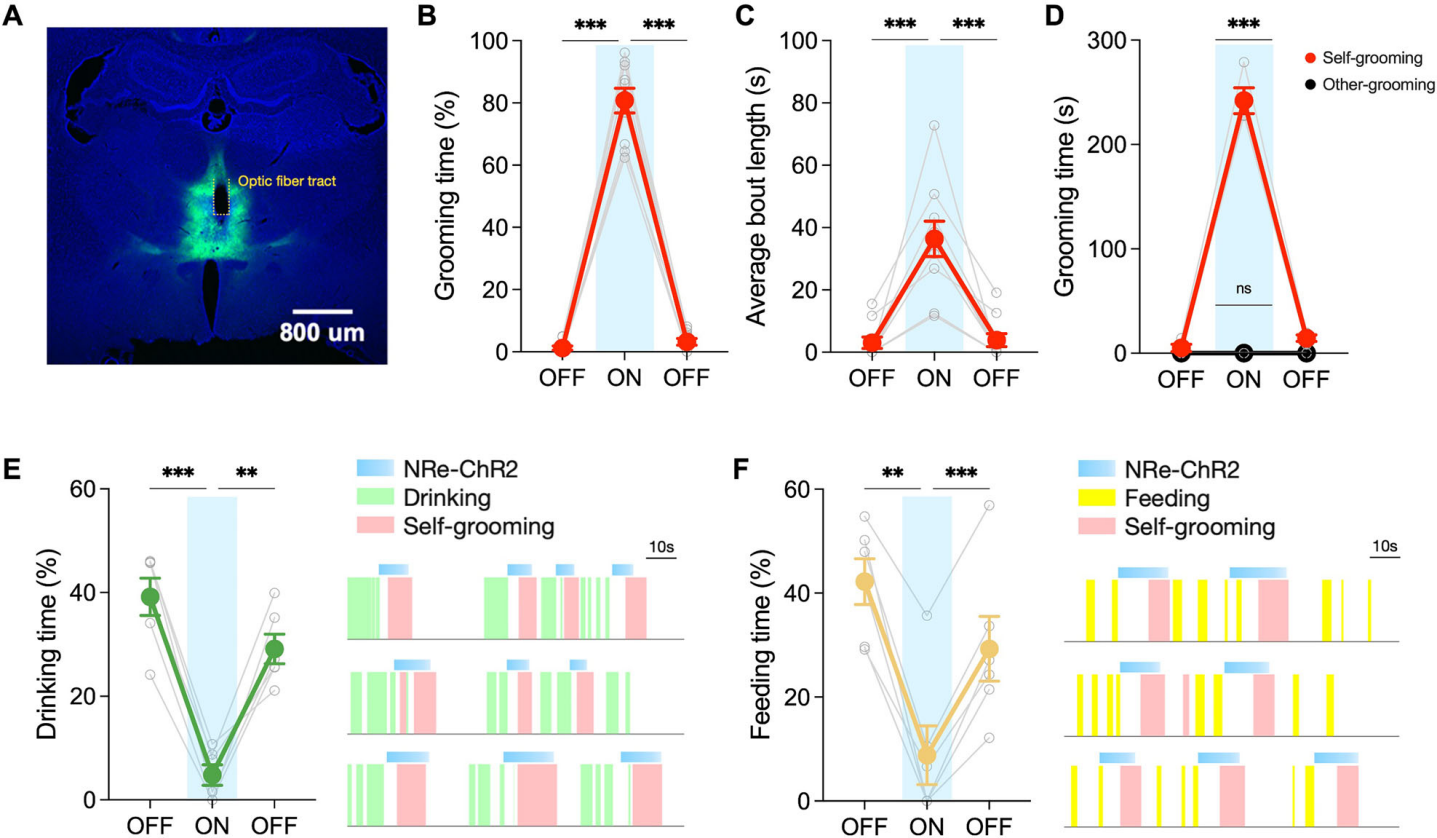
Optogenetic activation of nucleus reuniens triggers compulsive-like grooming behavior. **A** Localization of viral injection and optic fiber implant to achieve targeted optogenetic activation of NRe neurons expressing ChR2-eYFP. **B,** **C** Assessment of self-grooming behavior following ChR2-stimulation. NRe-activation led to a significant increase in (**B**) self-grooming duration and (**C**) the average length of a grooming bout. Repeated measures one-way ANOVA: effect of NRe stimulation on grooming duration: F(1.056, 9.501) = 389.6, *p* < 0.001; effect of NRe stimulation on average grooming bout length: F(1.006, 9.057) = 33.38, *p* = 0.0003. *n* = 10; Post-hoc Tukey HSD test to compare 1^st^ light-OFF, light-ON, and 2^nd^ light-OFF. **D** Rats demonstrated elevated self-grooming behavior during stimulation, but not allogrooming, in the presence of another conspecific. Repeated measures one-way ANOVA with post-hoc Tukey HSD test. *n* = 4. **E** Left panel: activation of NRe reduced drinking duration in water-restricted rats. Right panel: example raster plots showing optogenetic activation of NRe halted ongoing drinking behavior. Repeated measures ony-way ANOVA with post-hoc Tukey HSD test: F(1.190, 5.591) = 35.10, *p* = 0.0009. **F** Left panel: activation of NRe reduced feeding duration in food-restricted rats. Right panel: example raster plots showing optogenetic activation of NRe halted ongoing feeding behavior. Repeated measures ony-way ANOVA with post-hoc Tukey HSD test: F(1.198, 5.992) = 28.72, *p* = 0.0014; *n* = 6; **p < 0.01; ***p < 0.001. All data are presented as mean ± SEM.

To investigate the effects of NRe neuron activation, we utilized ChR2 to optogenetically stimulate these neurons in the absence of external grooming-inducing stimuli such as dirt or moisture (Fig. 2A). During 473-nm light stimulation of NRe, we observed that rats exhibited robust and repetitive grooming behavior, which persisted for over 80% of the stimulation duration, and ceased rapidly following the discontinuation of the light (*n* = 10 rats; Repeated measures one-way ANOVA: effect of NRe stimulation on grooming duration: F(1.056, 9.501) = 389.6, *p* < 0.001; 1^st^ light-OFF: 1.18 ± 0.66%; light-ON: 80.77 ± 3.96%; 2nd light-OFF: 3.21 ± 1.10%) (Fig. 2B). The duration of grooming episodes induced by NRe stimulation (36.36 ± 5.66 s) was significantly longer than spontaneous grooming bouts (3.02 ± 1.82 s), indicating the NRe’s role in driving persistent grooming behavior (Repeated measures one-way ANOVA: effect of NRe stimulation on average grooming bout length: F(1.006, 9.057) = 33.38, *p* = 0.0003) (Fig. 2C). Grooming in rodents is a socially relevant behavior, as rats often display grooming behavior not only on its own body (self-grooming) but also on that of their conspecifics (social grooming). Therefore, it is important to assess whether NRe-stimulation would also prompt social grooming, as a posterior thalamic-hypothalamic pathway has been recently discovered to be critical for social grooming in rodents [46]. Despite the presence of a conspecific, optogenetic stimulation of the NRe led exclusively to self-direct grooming behavior without any increase in social grooming (Fig. 2D).

To evaluate the intensity and compulsion-like nature of the grooming behavior induced by NRe activation, we examined the rats’ motivation for essential consumptive behaviors. Under water-restricted conditions, thirsty animals typically exhibit drinking behavior to reestablish fluid balance. However, upon activation of the NRe, the drinking behavior was significantly interrupted and rats initiated repetitive self-grooming instead (*n* = 6 rats; Drinking duration during 1st light-OFF: 39.19 ± 3.59%; light-ON: 4.82 ± 1.97%; 2nd light-OFF: 29.12 ± 2.86%; Repeated measures one-way ANOVA: effect of NRe stimulation on drinking duration: F(1.190, 5.591) = 35.10, *p* = 0.0009) (Fig. 2E). Water-drinking behavior was resumed only after the termination of NRe-activation, underscoring the disruptive potential of NRe hyperactivity. A similar pattern was observed with food intake; despite hunger due to food restriction, rats prioritized excessive self-grooming over feeding during NRe activation (Feeding duration during 1st light-OFF: 42.22 ± 4.41%; light-ON: 8.82 ± 5.67%; 2nd light-OFF: 29.28 ± 6.23%; Repeated measures one-way ANOVA: effect of NRe stimulation on feeding duration: F(1.198, 5.992) = 28.72, *p* = 0.0014) (Fig. 2F). These results demonstrate that NRe activation not only induces repetitive grooming behavior but does so with such intensity that it supersedes basic survival behaviors like drinking and eating, suggesting a powerful and possibly pathological influence of NRe on behavior.

### Aversiveness and anxiogenic effect of NRe activation

**Fig. 3:**
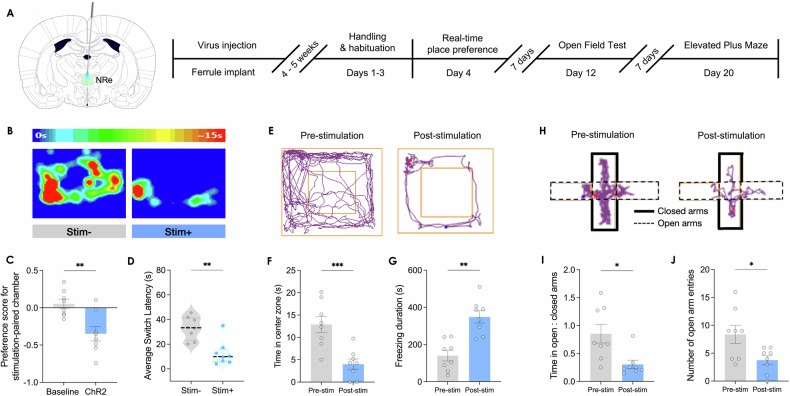
Activation of NRe induces place avoidance and anxiety-like phenotype. **A** Detailed experimental timeline for real-time place preference/avoidance assay, open field, and elevated plus maze experiments. **B** Representative heatmap results of real-time place preference/avoidance assay demonstrating rat spent less time in the chamber that was associated with optogenetic stimulation of NRe. **C** Quantification of rats’ occupancy of each chamber revealed a reduced preference for the stimulation-paired chamber during NRe-stimulation compared to the baseline condition (no ChR2-stimulation delivered). Wilcoxon matched-pair signed-rank test, *p* = 0.0078. **D** Upon entry to the ChR2-stimulation-paired chamber, rats showed a short latency of switching back to the light-unpaired chamber. Student’s paired two-tailed t-test: t(7) = 4.792, *p* = 0.0020. **E**–**G** Movement tracking results of open field test showing reduced time spent in the center one and increased freezing time after NRe-stimulation. Student’s paired two-tailed t-test. **H**–**J** Movement tracking results of elevated plus maze demonstrating reduced duration spent in open arms relative to closed arms and less open arm entries after NRe-stimulation. Student’s paired two-tailed t-test; *n* = 8; *p < 0.05; **p < 0.01; ***p < 0.001. All data are presented as mean ± SEM.

To better understand the affective implications of NRe activation, we drew parallels with the internal states reported by OCD patients, who often describe their compulsions as driven by distressing obsessions that paradoxically increase anxiety. We evaluated the affective valence associated with NRe photostimulation through a real-time place preference/aversion assay. NRe activation profoundly reduced the time rats spent in the stimulation-paired compartment, indicating aversion (*n* = 8 rats; Preference score for stimulation-paired chamber during baseline light-OFF phase: 0.055 ± 0.062; and during stimulation light-ON phase: −0.348 ± 0.097; Wilcoxon matched-pair signed-rank test: W = −36.00, exact *p* = 0.0078, two-tailed) (Fig. 3B, C). To ensure that this aversion was not simply a byproduct of NRe-induced increased grooming, we further analyzed the latency of rats to exit the light-paired compartment. Rats demonstrated a shorter latency to leave upon NRe activation (Average switch latency in the light-unpaired chamber: 32.99 ± 3.25 s; and in the light-paired chamber: 12.58 ± 3.53 s; Student’s paired t-test: t(7) = 4.792, *p* = 0.0020, two-tailed) (Fig. 3D), confirming that the photoactivation of NRe induced an avoidance phenotype that was independent of grooming behavior. Furthermore, as OCD patients often report elevated anxiety following compulsive behaviors, we investigated whether NRe activation could induce an anxiety-like state in rats by examining the degree of thigmotaxis – the tendency of a subject to remain close to walls in the open field test – which is well-validated to increase with rising anxiety levels in rodents [47, 48]. Indeed, after experiencing NRe-activation, rats exhibited an anxiety-like phenotype that was characterized by less time spent in the center zone (i.e., increased thigmotaxis) and increased freezing duration compared to the period before NRe-stimulation (Time spent in center zone of open field before NRe-stimulation: 12.91 ± 1.82 s; after NRe-stimulation: 4.01 ± 1.15 s; Student’s paired t-test: t(7) = 7.709, *p* = 0.0001, two-tailed) (Freezing duration before NRe-stimulation: 140.4. ± 28.5 s; after NRe-stimulation: 348.9 ± 33.3 s; Student’s paired t-test: t(7) = 5.392, *p* = 0.0010, two-tailed) (Fig. 3E–G). The elevated plus maze test, which exploits the general aversion of rats to open spaces, further corroborated these findings; rats showed a reluctance to explore open arms after NRe stimulation, spending less time and making fewer entries into the open arms (Ratio of time spent in open arms to closed arms of the elevated plus maze before NRe-stimulation: 0.855 ± 0.168; after NRe-stimulation: 0.305 ± 0.075; Student’s paired t-test: t(7) = 2.786, *p* = 0.0271, two-tailed) (Number of entries into open arms before NRe-stimulation: 8.38 ± 1.63; after NRe-stimulation: 3.75 ± 0.80; Wilcoxon matched-pair signed-rank test: W = −32.00, exact *p* = 0.0234, two-tailed) (Fig. 3H–J). These results demonstrate the aversive and anxiogenic effects of NRe photostimulation, suggesting that such neuronal activation is not merely a neutral trigger for compulsive behavior but is inherently distressing, akin to the negative affect and anxiety-like states commonly associated with OCD.

### Inhibition of NRe disrupted the maintenance of persistent self-grooming

**Fig. 4:**
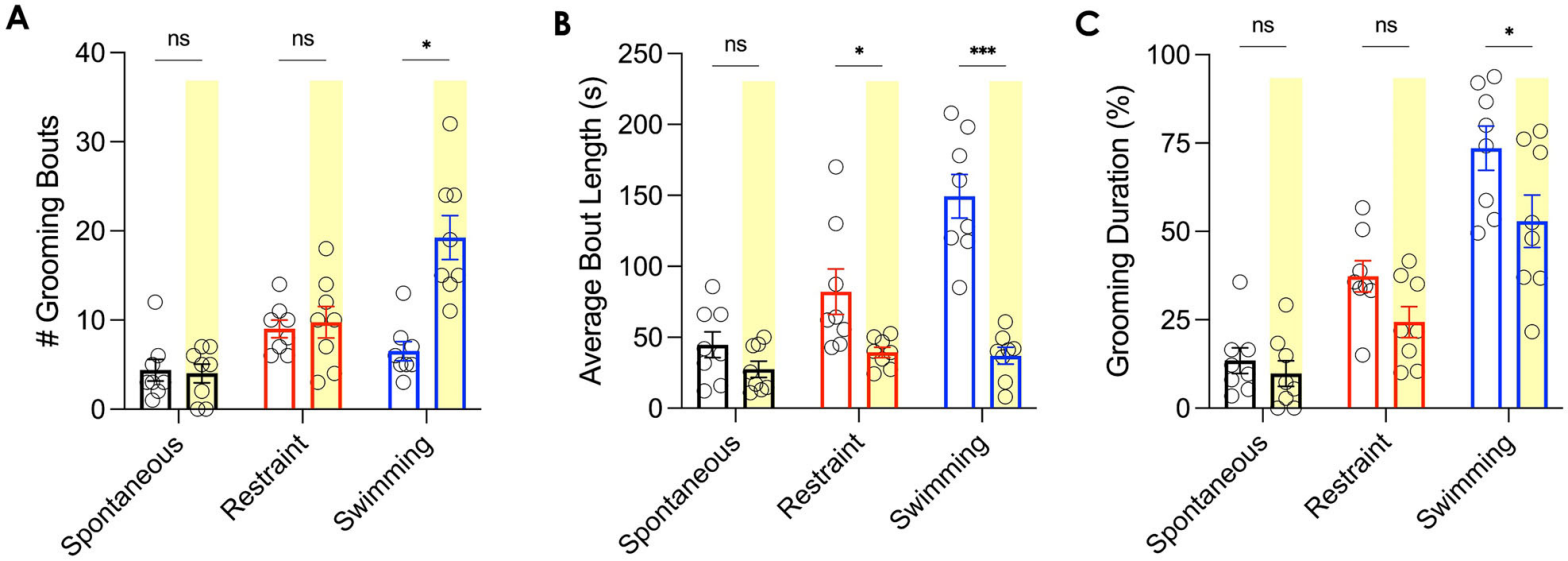
Inhibition of NRe disrupted the maintenachatnce of persistent self-grooming. **A,** **B** NRe-inhibition mainly altered the structure of swimming-induced grooming. Specifically, each bout was truncated into multiple, short bursts of grooming. **A** NRe-inhibition increased the number of grooming bouts under the swimming-induced group, but not the spontaneous or restraint-induced groups. Wilcoxon matched-pair signed-rank test, *p* = 0.0156. **B** NRe-inhibition resulted in shorter grooming bouts, especially in the swimming-induced context. Student’s paired two-tailed t-test: t(7) = 7.434, *p* = 0.0001. **C** The overall grooming duration was significantly attenuated in the swimming-induced group only. Wilcoxon matched-pair signed-rank test, *p* = 0.0234. *n* = 8; ns not significant; *p < 0.05; ***p < 0.001; All data are presented as mean ± SEM.

Furthermore, to establish a causal relationship between NRe activity and persistent self-grooming, we applied optogenetic inhibition by expressing eNpHR3.0 in NRe neurons and delivering a 589 nm laser under different grooming-inducing contexts. Interestingly, the primary effect of NRe-inhibition was demonstrated in the physical stress-induced context (i.e., fur-moistening through swimming), as indicated by an increase in the number of grooming bouts (*n* = 8 rats; light-OFF: 6.50 ± 1.07; light-ON: 19.25 ± 2.46; Wilcoxon matched-pair signed-rank test: W = 34.00, exact *p* = 0.0156, two-tailed) (Fig. 4A), and with each grooming bout lasting shorter (light-OFF: 149.5 ± 15.38 s; light-ON: 37.06 ± 5.91 s; Student’s paired t-test: t(7) = 7.434, *p* = 0.0001, two-tailed) (Fig. 4B). In other words, NRe-inhibition altered the structure of self-grooming induced by physical contamination, truncating what would typically be more prolonged bouts into multiple, short bursts of grooming. Moreover, the inhibition of NRe activity also led to an overall reduction of swimming-induced repetitive self-grooming behavior (light-OFF: 73.55 ± 6.22%; light-ON: 52.85 ± 7.41%; Wilcoxon matched-pairs signed rank test: W = −32.00, exact *p* = 0.0234, two-tailed) (Fig. 4C). However, this phenomenon was more pronounced in the physical stress-induced context than in the emotional stress (i.e., restraint) group. To elaborate, while optogenetic inhibition of NRe slightly reduced the average grooming bout length induced by restraint stress (light-OFF: 82.13 ± 16.03 s; light-ON: 39.44 ± 3.64 s; Student’s paired t-test: t(7) = 2.638, *p* = 0.0335, two-tailed) (Fig. 4B), it failed to elicit significant changes in the number of grooming bouts nor overall grooming duration. These findings suggest that the NRe is involved in both the generation and maintenance of persistent grooming behavior, particularly in contexts induced by physical contamination.

### A hypothalamic output of NRe contributing to excessive self-grooming

**Fig. 5:**
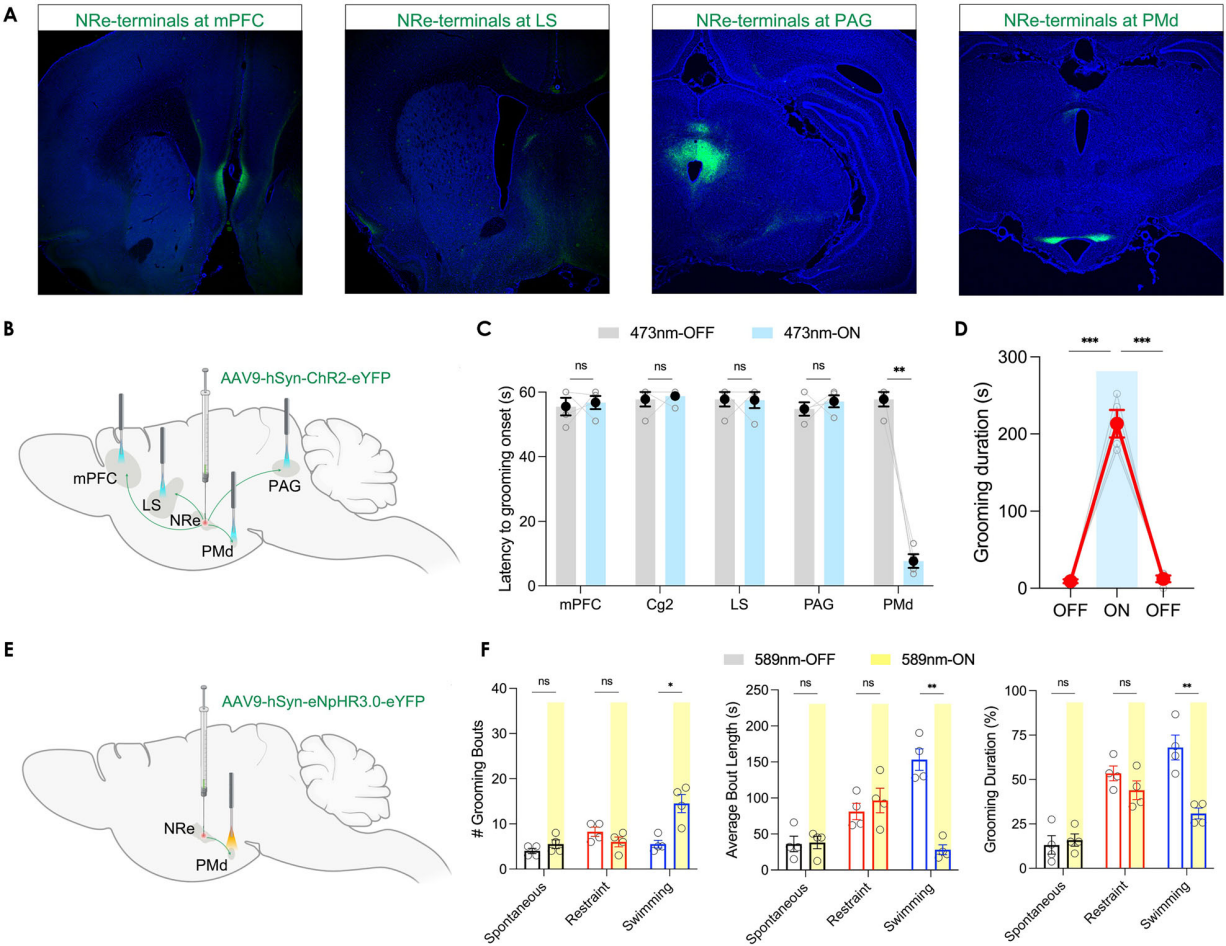
The NRe-PMd pathway contributes to persistent self-grooming behavior. **A** Anterograde tracing revealed projection from NRe to mPFC, PMd, lateral septum, and periaqueductal gray. **B** Activation of NRe-efferents by injecting AAV9-Syn-ChR2-eYFP into NRe and implanting optic fiber at the terminals of NRe. **C** Only optogenetic activation of terminal endings of NRe in PMd induced self-grooming behavior. Student’s paired two-tailed t-test: t(3) = 11.87, *p* = 0.0013. **D** NRe-PMd pathway activation led to a persistent grooming phenotype. Repeated measures one-way ANOVA with post-hoc Tukey HSD test: effect of NRe-PMd activation on grooming duration: F(1.032, 3.097) = 92.20, *p* = 0.0021; (**E**) Inhibition of NRe-efferents in PMd by injecting AAV9-Syn-eNpHR3.0-eYFP into NRe and implanting optic fiber at the PMd. **F** The optogenetic inhibition of NRe-PMd pathway only reduced grooming behavior in the swimming-induced group, but no the spontaneous or restraint-induced groups. Student’s two-tailed paired t-test: t(3) = 6.468, *p* = 0.0072. *n* = 4 for each group. ns not significant; *p < 0.05; **p < 0.01; ***p < 0.001. All data are presented as mean ± SEM.

Considering the extensive connections of the NRe with the cortico-hippocampal-thalamic network and the limbic system, we further explored the structure and function of NRe’s output circuitry. We injected AAV9-Syn-eGFP into the NRe, collected and analyzed the brains four weeks later to allow sufficient viral expression. The primary outputs of the NRe were identified by fluorescent signals in several brain areas, including the mPFC, the lateral septum, the periaqueductal gray, and the dorsal premammillary nucleus of the hypothalamus (PMd) (Fig. 5A). Recognizing that the diverse outputs of NRe neurons might be implicated in a broad spectrum of functions, we investigated the impact of each pathway on repetitive self-grooming behavior. To functionally activate the efferents of NRe, we injected AAV9-Syn-ChR2-eYFP into the NRe and implanted optic fibers into the downstream brain regions (Fig. 5B). Optogenetic activation revealed that only the NRe-PMd pathway could robustly induce repetitive self-grooming behavior, characterized by a rapid onset of grooming behavior upon stimulation (Latency to grooming onset before light stimulation: 5.75 ± 2.25 s; after light stimulation: 7.70 ± 2.11 s; Student’s paired t-test: t(3) = 11.87, *p* = 0.0013, two-tailed) (Fig. 5C). Similar to NRe-stimulation, activation of the NRe-PMd pathway robustly induced prolonged repetitive grooming behavior in rats, which ceased rapidly after the cessation of light stimulation (Repeated measures one-way ANOVA: effect of NRe-PMd activation on grooming duration: F(1.032, 3.097) = 92.20, *p* = 0.0021; 1^st^ light-OFF: 9.15 ± 2.43 s; light-ON: 213.3 ± 17.88 s; 2^nd^ light-OFF: 12.23 ± 4.36 s) (Fig. 5D). Furthermore, to investigate whether pathway-specific inhibition of NRe-PMd efferents could reduce grooming behavior, we injected AAV9-Syn-eNpHR3.0-eYFP into the NRe and implanted optic fibers into its efferents at PMd (Fig. 5E). Remarkably, the inhibition of the NRe-PMd pathway only modulated grooming behavior induced by physical contamination (i.e., swimming), but leaving spontaneously occurring and restraint stress-induced grooming behavior unaltered. To illustrate, the effects of this pathway-specific inhibition were manifested as largely truncated grooming bouts and an overall significant reduction of grooming induced by swimming only (number of grooming bouts during light-OFF: 5.50 ± 0.87; light-ON: 14.50 ± 2.02; average duration of each grooming bout during light-OFF: 153.3 ± 14.96 s; light-ON: 28.18 ± 6.83 s; overall grooming duration during light-OFF: 68.05 ± 6.98%; light-ON: 30.90 ± 3.03%; Student’s paired t-test: t(3) = 6.468, *p* = 0.0075, two-tailed) (Fig. 5F). These observations hence suggest a critical role of this midline thalamic-hypothalamic pathway in regulating specifically physical contamination-related repetitive grooming behavior.

## Discussion

The nucleus reuniens, a component of the ventral midline thalamic nuclei, is implicated in cognitive flexibility, fear memory extinction, and the inhibition of perseverative behavior [49]. These functions are notably deficient in patients with OCD, and the severity of OCD symptoms has been linked to abnormal neuronal transmission within the midline thalamus [50]. Our study investigates the role of the NRe in OCD-like compulsive behaviors in rats, expanding the current research focus beyond the frontal cortices and striatum. Despite the inherent limitations of evaluating thought content in non-human animals while addressing the obsessive component of OCD [51], our findings revealed a previously overlooked role of the NRe in producing OCD-like behavioral manifestations, specifically excessive self-grooming in rats—a behavior that resembles compulsive handwashing in OCD patients [52, 53].

Rodent self-grooming is a well-established, frequently occurring, and innate behavior associated with cleanliness, thermoregulation, and stress reduction [41]. This behavior is also seen in humans and can become pathological in neuropsychiatric disorders, including OCD [54]. Our study reveals that activation of NRe robustly leads to a persistent self-grooming phenotype with excessive time spent performing a ritualistic grooming action to the point of disrupting survival-essential behaviors like eating and drinking, irrespective of hunger or thirst. This finding is distinct from existing literature, which typically reports only an overall increase in grooming frequency or duration but not its persistence [38, 55].

Our data suggest that NRe photostimulation not only triggers compulsive-like grooming but also associates this behavior with negative emotional valence in the real-time place preference/avoidance assay. This indicates an overwhelming urge to groom despite aversive stimuli, a finding that contrasts with optogenetic models of autism spectrum disorder where photostimulation relates to positive valence [38, 56, 57]. Additionally, post-stimulation anxiety-like behaviors in rats mirror the distress OCD patients experience when compulsively acting on obsessions, which does not alleviate—but often paradoxically increases—their anxiety. Importantly, optogenetic inhibition of the NRe not only decreased overall grooming time but also significantly disrupted the persistent grooming pattern in the context of physical stress-induced grooming. This suggests the NRe’s involvement not only in the initiation but also in the maintenance of OCD-like grooming, mainly when triggered by physical contamination.

Our study has pinpointed the PMd, a downstream area of NRe, as a key area in the regulation of compulsive-like grooming behaviors. The PMd is a critical component of the medial hypothalamic defensive system, orchestrating responses to predator threats, such as exposure to a wild cat’s urine odor [58–61]. This defensive role offers a valuable perspective for understanding why rats exhibit excessive grooming when the NRe is stimulated. It suggests that compulsive grooming may be an extension of the PMd’s defensive repertoire, acting as a coping mechanism in response to perceived threats. The rapid onset of grooming behaviors following NRe-PMd pathway activation aligns with previous findings where other brain regions, like the paraventricular hypothalamus and the orbitofrontal-striatal projection, have been stimulated to induce similar repetitive actions [11, 62]. This type of excessive grooming may serve as a defensive coping response, comparable to how obsessions in OCD patients represent a response to perceived existential threats [63, 64]. It is possible that such grooming behaviors, while initially serving as coping strategies, could become maladaptive and pathological through chronic and repeated abnormal neuronal activity in this thalamic-hypothalamic pathway, potentially contributing to compulsive behaviors characteristic of OCD. Given these observations, our findings underscore the need for further investigation into how chronic hyperactivity in this pathway may be implicated in OCD, thereby offering new avenues for potential therapeutic interventions.

The paraventricular nucleus (PVN) of the hypothalamus is known to play a pivotal role in the regulation of both grooming and stress responses [41, 65, 66]. Our results contribute to this understanding by showing c-Fos expression in the PVN under different stress-induced grooming models. While the PVN has been classically associated with regulating neuroendocrine stress responses and grooming driven by homeostatic mechanisms [57, 65, 67], our findings extend beyond these classical associations by illustrating that the NRe may facilitate a specific type of excessive grooming that carries a negative affective valence, similar to the compulsions exhibited in OCD. Not only did we observe increased c-Fos expression in the NRe, which hints at its potential role in grooming, but we also successfully elicited grooming behaviors through optogenetic activation of the NRe without stimulating the PVN. The grooming evoked by NRe-stimulation also differed from that induced by the PVN in terms of the rapid onset and prolonged duration [57, 62]. Furthermore, the direct projection from the NRe to the PMd, which is responsible for coordinating defensive behaviors [61], positions the NRe distinctly from the PVN, which predominantly influences grooming via neuroendocrine pathways [65]. This distinction suggests that while the PVN is involved in grooming related to homeostatic restoration, the NRe may drive compulsive grooming as a maladaptive coping mechanism. However, the relationship between the PVN and NRe is likely complex and not entirely segregated. The PVN has some level of connectivity with both the NRe and PMd [68, 69], which implies that there could be an interplay between these regions that influences the shift from adaptive to compulsive grooming behaviors. Future research dedicated to mapping the functional connections between these regions during various grooming scenarios could offer further insights into the underpinning mechanisms. Furthermore, while the current study only focuses on contamination cleaning-related OCD symptoms in male animals, it would be highly valuable to include females as well as examine other symptom dimensions of OCD, including checking, symmetry precision, and hoarding behaviors to obtain a comprehensive picture of OCD etiology and pathophysiology [70–72].

In summary, our findings indicate that the NRe plays a central role in mediating excessive self-grooming behavior that is both aversive and disruptive. This behavior bears a strong resemblance to the compulsive behaviors observed in OCD, such as excessive handwashing. This relationship is likely due to the NRe’s strategic position within the extensive cortico-hippocampal-thalamic network that regulates cognition, emotion, and stress-related behaviors. Consequently, these insights suggest that the NRe could be a potential target for interventions aimed at mitigating OCD-related compulsive behaviors.

## Data Availability

The data that support the findings of this study are available from the corresponding author upon reasonable request.
